# AlPOs Synthetic Factor Analysis Based on Maximum Weight and Minimum Redundancy Feature Selection

**DOI:** 10.3390/ijms141122132

**Published:** 2013-11-08

**Authors:** Yuting Guo, Jianzhong Wang, Na Gao, Miao Qi, Ming Zhang, Jun Kong, Yinghua Lv

**Affiliations:** 1 College of Computer Science and Information Technology, Northeast Normal University, Changchun 130117, Jilin, China; E-Mails: guoyt484@nenu.edu.cn (Y.G.); kongjun@nenu.edu.cn (J.K.); 2 Faculty of Chemistry, Northeast Normal University, Changchun 130024, Jilin, China; 3 Key Laboratory of Intelligent Information Processing of Jilin Universities, Northeast Normal University, Changchun 130117, Jilin, China; E-Mail: qim801@nenu.edu.cn; 4 State Key Laboratory of Inorganic Synthesis and Preparative Chemistry, Changchun 130012, Jilin, China; E-Mail: gaona0431@163.com

**Keywords:** AlPOs, data mining, feature selection, rational synthesis

## Abstract

The relationship between synthetic factors and the resulting structures is critical for rational synthesis of zeolites and related microporous materials. In this paper, we develop a new feature selection method for synthetic factor analysis of (6,12)-ring-containing microporous aluminophosphates (AlPOs). The proposed method is based on a maximum weight and minimum redundancy criterion. With the proposed method, we can select the feature subset in which the features are most relevant to the synthetic structure while the redundancy among these selected features is minimal. Based on the database of AlPO synthesis, we use (6,12)-ring-containing AlPOs as the target class and incorporate 21 synthetic factors including gel composition, solvent and organic template to predict the formation of (6,12)-ring-containing microporous aluminophosphates (AlPOs). From these 21 features, 12 selected features are deemed as the optimized features to distinguish (6,12)-ring-containing AlPOs from other AlPOs without such rings. The prediction model achieves a classification accuracy rate of 91.12% using the optimal feature subset. Comprehensive experiments demonstrate the effectiveness of the proposed algorithm, and deep analysis is given for the synthetic factors selected by the proposed method.

## 1. Introduction

As an important class of crystalline materials, zeolites and related microporous materials have been widely used in the petroleum industry for catalysis, separation and ion-exchange [1,2]. Following the discovery of the aluminophosphate molecular sieves AlPO_4_-*n* (*n* denotes the structure type) in 1982, a large variety of open-framework aluminophosphates with different structure types have been synthesized and open-framework aluminophosphate materials has become an important member of the porous crystal material family. Recently, the rational synthesis of microporous inorganic materials has attracted extensive attention [3–10]. However, since the synthesis of such materials is typically carried out in a gel medium under hydrothermal/solvothermal conditions by using alkali metal ions or organic amines/ammoniums as the templates or structure-directing agents (SDAs) [11], it is very complicated and influenced by many factors. Therefore, in order to provide guidance to rational synthesis of microporous inorganic materials, the researchers of State Key Laboratory of Inorganic Synthesis and Preparative Chemistry of Jilin University established an international ALPO synthesis database [12] based on a large number of synthesis experiments and collections from the papers.

With the rapid development of computer technology and artificial intelligence, data mining plays an increasingly important role in more and more research areas. The goal of data mining is to find the implied knowledge from the given data. The applications of data mining techniques in chemical science have shown their feasibility for numeric calculation, simulation and data analysis. Nowadays, one of the most widely used data mining techniques in chemical science is feature selection. Feature selection is usually used as a preprocessing step in machine learning that can select the most important features for particular tasks by seeking the potential information hidden in the data. Recently, several feature selection methods were successfully applied in chemical data analysis. Pichler [13] developed an interactive feature selection method based on KNN (K Nearest Neighbor) to classify doublet/singlet patterns from the same Stationary Electrode Polarography (SEP) data. Liu evaluated the performance of the methods as Information Gain, Mutual Information, χ^2^-Test (CHI), Odds Ratio (OR) and GSS Coefficient (GSS) for finding the optimal feature subset in drug discovery; the features were firstly ranked according to the scores obtained by different feature selection methods and then the top-ranking features were used for classification task [14]. Teramoto and Fukunishi proposed a supervised consensus scoring (SCS) method for docking and virtual. In SCS, a series of scoring functions including PLP, *F*-Score, LigScore, DrugScore, LUDI, *X*-Score, AutoDock, PMF, *G*-Score, ChemScore and *D*-Score were integrated to form a complementary scoring function, which could compensate for the deficiencies of each scoring method [15]. In addition, a Mutual Information Gain algorithm was utilized to generate a feature subset which excluded features having weak correlation with the target variable, and then the selected features were input into a Genetic Programming model to analyze QSAR (Quantitative Structure Activity Relationship) data [16]. In a further study, 649 bitter and 13,530 randomly selected molecules from the MDL Drug Data Repository (MDDR) were analyzed by Information Gain, and the selected features were then classified by Naive Bayes classifier to identify the bitterness of small molecules [17]. Feature selection methods also have been applied to AlPOs database analysis. Li *et al*. evaluated the classification performance produced by different combinations of synthetic features (11 features in total) using Support Vector Machines (SVM), and then checked which individual or combined features effected most for distinguishing the two classes of AlPOs. They found that suitable template parameters were of vital importance to the classification performance [18]. Huo *et al*. [19], measured the importance of the various synthetic features (26 features in total) of AlPOs by *F*-Score [20], and sorted the features in descending order according to their importance degree. The features were then added into Decision Tree (DT) model orderly to test their discriminative abilities. They regarded the feature subset that could reach the best classification performance as the optimal subset. Through their experiments, they found that T1_Distance2 (the second longest distance of organic template) was the determinant factor to distinguish AlPO_4_-5 from other types of aluminophosphate molecular sieves. Although the pioneering works in [18] and [19] have shown that the feature selection techniques can be applied for AlPOs database analysis effectively, there were also some limitations in them. Firstly, the feature subset evaluated in [18] was generated using an exhaustive searching strategy, which made it hard to be scaled to high-dimensional AlPOs data. Secondly, the optimal feature subsets in both [18] and [19] were evaluated by a specific classifier (DT in [19] and SVM in [18]). Thus, the classifiers need to be trained and tested many times in the feature selection procedure, which made them very time-consuming. Finally, the correlation among the selected features was neglected in both [18] and [19]. Some researchers [21] have pointed out that a good feature subset should be the one that contains features highly correlated with the class, while uncorrelated with each other. Therefore, ignoring the correlation among the selected features might cause the problem of “information redundancy”, which hinders optimal results from the selected features.

In order to overcome the limitations of the previous works, a new feature selection algorithm based on maximum weight and minimum correlation criterion is proposed in this paper. The proposed method not only considers the importance of the feature, but also takes the correlation among the selected features into account. Thus, through the proposed method, we can select the optimal feature subset in which the features are maximally relevant to the synthetic structure while the redundancy among these selected features is minimal. In the experiments, three feature evaluation algorithms (Fisher score, ReliefF score and Gini score) are combined with redundancy measurement method (Pearson correlation coefficient) to test the performance of our method. Compared with other feature selection methods [18,19] for AlPOs database analysis, our method possesses the following advantages. (1) The optimal feature subset generated by our method does not depend on any classifier. Thus, the feature selection procedure does not need to train any classifier, which makes our algorithm more efficient; (2) The feature selection procedure of our algorithm is a pair-wise updating optimization process, so it can be easily scaled to high-dimensional AlPOs data; (3) The proposed method takes the correlation among features into consideration. Thus, it can obtain better results than other state of the art feature selection methods.

## 2. Results and Discussions

In this section, we first compare the performance of the proposed algorithm with other classical scoring feature selection methods that neglect the correlation among features during the feature selection process. Then, the feature selection results obtained by the proposed algorithm are analyzed and compared with the previous works [18,19]. At last, we also compare the proposed algorithm with several state of the art feature selection approaches, such as Constraint score [22], MRMR [23,24] and FCBF [25].

In the experiments, the Nearest Neighbor and Naive Bayes classifiers are employed as prediction models for their advantage of simplicity. In order to validate the effectiveness of the algorithm comprehensively, we use 10-fold cross validation in the experiments.

### 2.1. Performance Measures

The synthetic records used in the experiments contain 398 (6,12)-ring-containing AlPOs and 852 AlPOs without such rings. For the purpose of distinguishing the (6,12)-ring-containing AlPOs from others, we deem the former as positive samples and the latter as negative samples respectively. It is obvious that the numbers of positive and negative samples are imbalanced in this study. So besides the classification accuracy rate, we also utilize the *F*-measure to evaluate the performances of the proposed algorithm.

Suppose *n_+_* and *n_−_* are the numbers of positive samples and negative samples. With reference to the confusion matrix [26] in Table 1, the classification accuracy rate (Acc_Rate) and *F*-measure can be denoted as:

(1) $$\text{classification accuracy rate}=\frac{TP+TN}{n_{+}+n_{-}}$$

and

(2) $$F-\text{measure}=\frac{(1+\beta^{2})\text{recall}\times \text{precision}}{\beta \times \text{recall}+\text{precision}}$$

where recall = *^TP^*/*_TP_*_+_*_FN_*, precision = *^TP^*/*_TP_*_+_*_FP_*, and β is a parameter to adjust the relative importance degree between recall and precision. In this work, we set β as 1. The value of *F*-measure lies between 0 and 1, with value closer to 1 indicating better performance for imbalanced problems.

### 2.2. Effectiveness of the Proposed Method

**Figure 1:**
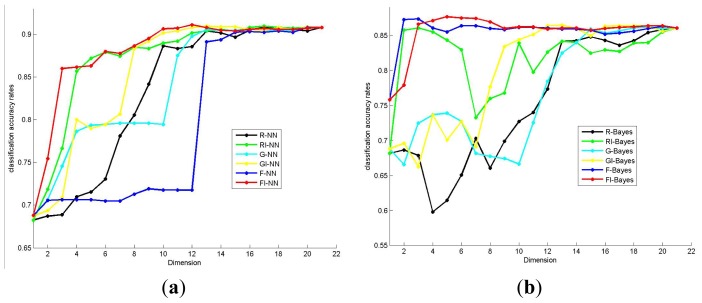
Comparison of the original and proposed feature selection methods. (**a**) Using Nearest Neighbor as classifier; (**b**) Using Naive Bayes as classifier.

We will firstly verify the effectiveness of the proposed method by comparing it with some classical scoring feature selection methods without considering the correlation among features. In this experiment, Fisher score [27], ReliefF score [28] and Gini score [29] are applied to measure the importance of the feature, while Pearson Correlation Coefficient (PCC) is applied to measure the correlation among features. The classification accuracy rate of different methods under various feature dimensions can be seen in Figure 1. In this figure, FI (Fisher improve) denotes the proposed method that utilizes the Fisher score and Pearson Correlation Coefficient to estimate the importance and correlation of features. RI (ReliefF improve) denotes the proposed method that utilizes the ReliefF score and Pearson Correlation Coefficient to estimate the importance and correlation of features. And GI (Gini improve) denotes the proposed method that utilizes the Gini score and Pearson Correlation Coefficient to estimate the importance and correlation of features. *F*, *R* and *G* denote original Fisher score, ReliefF score and Gini score respectively. The best classification accuracy rates obtained by these methods are listed in Table 2.

From Figure 1 and Table 2, it can be seen that through taking the correlation among the selected features into consideration, the proposed algorithm can outperform the classical scoring feature selection methods. When the Nearest Neighbor classifier is utilized, the best classification accuracy rates obtained by FI, RI and GI are 91.12%, 90.96% and 90.96% respectively. When the Naive Bayes classifier is employed, the best classification accuracy rates obtained by FI, RI and GI are 87.67%, 86.08% and 86.48%. Moreover, it also should be noted that the dimensions of the optimal feature subset obtained by the proposed algorithm are less than the classical scoring feature selection algorithms in most cases.

In this paper, the numbers of the positive samples and negative samples are imbalanced, so we take the *F*-measure to evaluate the performance of proposed method. As shown in Table 3, the proposed algorithm is superior to the classical scoring feature selection methods for the class imbalance problem especially when the Fisher score is utilized to evaluate the importance of feature.

From above experimental results, we can find that the performance of Fisher score combined with PCC in the proposed algorithm is superior to ReliefF score and Gini score combined with PCC, since the optimal feature subset generated by FI is in a lower dimension and wins the highest classification accuracy rate as well. So in the next experiment, we will focus on analyzing the features selected by FI.

### 2.3. Analysis of the Feature Selection Results

In this part, we make some analysis about the feature selection result obtained by the proposed method (Fisher score combined with PCC) and compare our feature selection result with the previous works [18,19].

Here, let’s reconsider the performances of the proposed method (FI-NN) and the original Fisher score (F-NN) in Figure 1a. Firstly, we can find that the classification performances of the proposed method are superior to the original Fisher score under most dimensions. This means that by reducing the redundancy from selected features, the proposed method can select more optimal feature subsets for distinguishing the (6,12)-ring-containing AlPOs. Secondly, it can be observed that when the dimension of the selected features increases from 1 to 12, the classification performance of the FI shows a dramatic upward trend, and reaches its peak point at dimension 12. However when the dimensions of the selected features are larger than 12, the classification performance presents a tiny downward trend. This suggests that the features selected in the previous 12 dimensions may take significant information for separating the (6,12)-ring-containing AlPOs from others. Thirdly, classification accuracy rate sharply increases from about 75.5% to 86.2% when F12 (the second longest distance of organic template) is added to the optimal feature subset at dimension 3. This phenomenon indicates that the second longest distance of organic template is a very crucial factor for the rational synthesis of (6,12)-ring-containing AlPOs.

**Figure 2:**
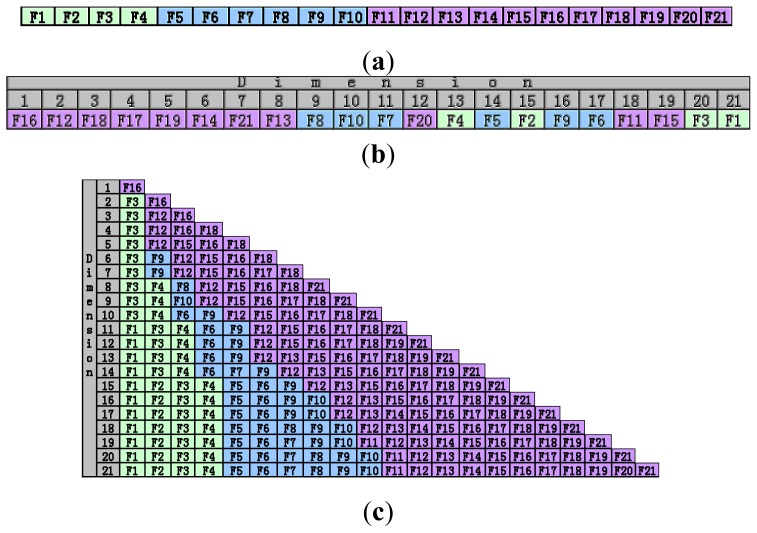
The features selected by Fisher and Fisher combined with PCC. (**a**) Different category synthesis factors are represented as different color; (**b**) features selected by Fisher score; (**c**) features selected by Fisher score combined with PCC in our algorithm.

Figure 2 shows the feature selection results obtained by Fisher score and the proposed method (FI). In this figure, in order to distinguish different types of features more clearly, we assign different colors to different categories of features (as shown in Figure 2a). The features marked with green color belong to gel composition, the features marked with blue color and purple color belong to solvent and organic template. Figure 2b,c illustrate feature subsets selected by the original Fisher score and the proposed algorithm (FI). In Figure 2b, the features are sorted in descending order according to their Fisher scores. Since the features with higher Fisher scores are more important, if we want to obtain a feature subset that contains *k* features, we just need to select the first *k* features in the descending sequence mentioned above. Figure 2c demonstrates the features selected by the proposed algorithm (FI) under every dimension (the selected features are sorted in ascending order according to their ID). The features selected by FI under dimension 12 which could lead to the highest classification accuracy rate are F1 (the molar amount of Al_2_O_3_), F3 (the molar amount of solvent), F4 (the molar amount of template in the gel composition), F6 (the melting point), F9 (the dipole moment), F12 (the second longest distance of organic template), F15 (the dipole moment), F16 (the ratio of *C/N*), F17 (the ratio of *N*/(*C* + *N*)), F18 (the ratio of *N*/Van der Waals volume) and F21 (the maximal number of protonated H atoms).

There is a remarkable phenomenon in Figure 2b that the first 8 features selected by Fisher score are all marked with purple color, which means they all belong to organic template. Although the organic template factors are significant for AlPOs synthesis, these factors are not sufficient to distinguish AlPOs with different structures effectively. From Figure 1a, we can find that when the first two features F16 (ratio of *C/N*) and F12 (second longest distance of organic template) are selected, the classification accuracy rate of the classifier could reach about 71%. However, after the other 6 template features with higher Fisher score (F18 (the ratio of *N*/Van der Waals volume), F17 (the ratio of *N*/(*C* + *N*)), F19 (the Sanderson electronegativity), F14 (the Van der Waals volume), F21 (the maximal number of protonated H atoms), F13 (the shortest distance of organic template)) are added into the selected feature subset gradually, the classification accuracy rate of the classifier is almost unchanged. Failure of the feature selection described above is caused by information redundancy, or the correlation among the selected features. Since the first 8 features in Figure 2b come from the same category (organic template), they are far from orthogonal and cannot improve the performance of the classification task. In other words, although the first 8 template features in Figure 2b have higher Fisher score values, selecting them all into the feature subset does not enable the addition of new information into the selected feature subset. This clarifies the importance of accounting for redundancy during the feature selection process.

Li *et al*. found the optimal feature subset was consisted of 8 features that obtained the highest classification accuracy rate of 82.44% by SVM classifier [18]. However, their feature selection process was an exhaustive searching strategy, so the entire process was extraordinarily time consuming. In their study, the correlation among the selected features was not at all mentioned. Thus, the correlations between some of the selected features were very high, for example: the correlation between F7 (boiling point) and F8 (dielectric constant) was 0.8370; the correlation between F7 (boiling point) and F9 (dipole moment) was 0.8306; the correlation between F8 (dielectric constant) and F9 (dipole moment) was 0.9512. Huo *et al*. worked out that a feature subset consisting of 19 features was the best combination for predicting AlPOs, with the highest *AUC* of 90% and the highest classification accuracy rate of 88.18% [19]. Nevertheless, since the correlation among features was also neglected in their study, there were some highly correlated features in their optimal feature set too. For example, the correlation between F8 (dielectric constant) and F10 (polarity) was 0.9849, while the correlation between F14 (Van der Waals volume) and F20 (number of free rotated single bond) was 0.9073.

In the proposed method, we take into account the correlation among the selected features in the feature selection process. So, as shown in Figure 1a the classification accuracy rate curve of FI presents a distinctive uptrend before getting to the peak point, and when features belonging to a new category are added to the selected feature set at dimension 2 and 6, the curve appears obviously ascending. In the optimal feature set produced by this study, the molar amount of Al_2_O_3_, solvent and template are gel composition features; melting point and dipole moment are solvent features; the second longest distance of organic template, the dipole moment, the ratio of *C/N*, the ratio of *N/*(*C* + *N*), the ratio of *N*/Van der Waals volume and the maximal number of protonated H atoms are organic template features. Since the selected features by the proposed algorithm are comprehensive, we obtain the highest classification accuracy rate as 91.12% using Nearest Neighbor classifier, which is much simpler than the classifiers employed in [18] and [19]. Xu *et al*. pointed out that synthesis of microporous aluminophosphate was carried out in a gel medium under hydrothermal/solvo-thermal conditions by using the templates as structure-directing agents [11]. Gel composition is the material basis for producing chemical reaction, solvent provides the reaction environment, and template plays a role of structure-directing. Among the optimal features, F12 (second longest distance of organic template) is the most important feature. In the rational synthesis of microporous materials, the geometric factor of the organic template plays a vital role to affect the shape and the pore size of an AlPO structure. For open-framework AlPOs with (6,12)-rings, the organic templates are usually located in the one-dimensional 12-ring channels, thus their longest direction is extended along the channels. Therefore, the second longest distance of the organic templates is determinative to the window size of the channels [18]. From Figure 2, we can see that the optimal feature subset selected by our method contains 12 features belonging to three categories. However, the original Fisher score only selected features from two categories at dimension 12. Moreover, we can find that the second longest distance of the organic templates (F12) is selected by our method. These observations indicate that the proposed method is more consistent with the prior knowledge of synthetic chemists.

Compared with the methods in previous works [18,19], the proposed method has the following advantages. Firstly, it is independent of any classifier. Thus, as can be seen from the experimental results, the performances of our method are superior to other classical feature selection algorithms under both Nearest Neighbor and Naive Bayes classifiers. Secondly, the proposed method takes the correlations among the selected features into consideration. Therefore, it can remove the redundant information from the selected feature subset. However, we should point out that there also exists an inconvenient point in the proposed method. Since various feature scoring and correlation measurement algorithms can be incorporated into our method, there may be a need to conduct experiments to verify which combination of feature scoring and correlation measuring algorithms can obtain the best feature selection result.

### 2.4. Comparisons with Other Feature Selection Methods

**Figure 3:**
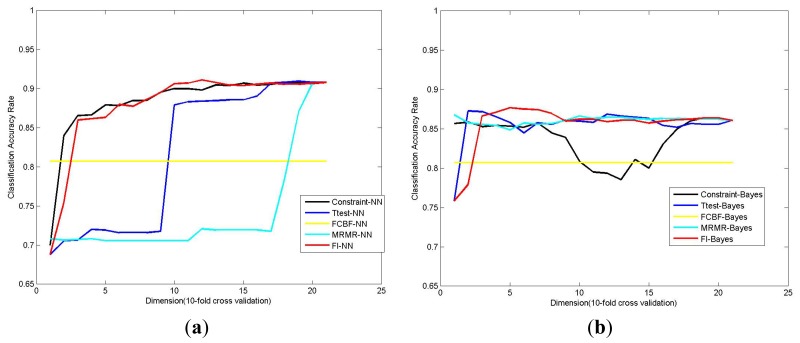
Performance comparison of the proposed algorithm and some popular feature selection methods. (**a**) Using nearest neighbor as classifier; (**b**) Using Naive Bayes as classifier.

In this subsection, we compare the performance of the proposed method with some other state of the art feature selection methods including *T*-test [30], Constraint score [22], MRMR [23,24] and FCBF [25]. Among these methods, *T*-test and Constraint score are univariate feature selection methods that select features by the weights or importance degrees of features, while both FCBF and MRMR are multivariable feature selection methods that take the correlation among the selected features into consideration. We compare their performances under various dimensions on the AlPOs dataset (Figure 3). Here, it should be noted that since the number of selected features cannot be predefined in FCBF, we are unable to test its performance under every dimension. Thus, only the average classification accuracy rate of 10-fold cross validation of FCBF is shown in Figure 3. The best classification accuracy rates obtained by these methods are listed in Table 4.

From Figure 3 and Table 4, we can find that the proposed algorithm outperforms other feature selection methods since it could get higher classification accuracy rate under relatively lower dimension, especially when the Nearest Neighbor is utilized for classification. However, it also can be observed that the proposed algorithm does not win over other algorithms by a very large margin in some cases. Therefore, like the experiments in Section 2.2, the *F*-measure is also employed here to evaluate the performances of different algorithms. From the *F*-measure values obtained by different algorithms in Table 5, we can see that the performance of the proposed algorithm is much better than other algorithms. These experimental results are consistent with Section 2.2.

## 3. Materials and Method

### 3.1. Data Sets

The microporous aluminophosphate dataset used in this paper comes from the database of AlPOs synthesis established by the State Key Laboratory of Inorganic Synthesis and Preparative Chemistry of Jilin University (http://zeobank.jlu.edu.cn/). This database contains 1600 synthetic records in all. After removing the records that contain missing values (about 29% of the total), we use the remainder 1250 records in our experiment. In these records, 398 (6,12)-ring-containing AlPOs are deemed as positive samples, while 852 non-(6,12)-ring-containing AlPOs are deemed as negative samples. In this study, 21 synthetic features (or factors) belonging to three categories (Gel composition, Solvent and Organic template) are concerned (shown in Table 6). For more details about the definitions and meanings of the synthetic factors in Table 6, see [31].

### 3.2. The Proposed Algorithm

Formally, suppose *D* = [*d*_1_, *d*_2_,..., *d_n_* ]ε *R^m×n^* is the input dataset that contains *n* samples in *m* dimensional space (For the microporous aluminophosphate dataset utilized in this study, the values of *m* and *n* in *D* are 21 and 1250, respectively). We can denote each row vector of *D* by *P_i_* (*i* = 1, …, *m*), which is corresponding to a feature. The aim of the proposed feature selection algorithm is to select *k* (*k* < *m*) features from the original feature set to form a feature subset *U* in which the importance of the features are maximizing and the correlations among the features are minimizing.

Let *S* = [*s*_1,_*s*_2,_…,*s_m_*]*^T^* ε *R^m^*^×1^ be the positive weight of each feature which reflects its importance, where *s_i_* is the weight of the *i*th feature (*i* = 1, …, *m*). In this study, the weights of features can be obtained by any classical feature evaluation method (such as Fisher score, ReliefF score and Gini score), and the features with larger weights are more important. Let*C* ε *R^m^*^×^*^m^* be the correlation matrix, where *C_ij_* ≥ 0(*i*≠ *j*) indicates the correlation between the *i*th and *j*th features. Since the self-correlation of the synthetic factor is meaningless, we assign the diagonal elements *C_ii_* (*i* = 1, 2, …, *m* ) to be 0. *f* = [ *f*_1_, *f*_2_,..., *f_m_*]*^T^* is an indicator vector, where *f_i_* = 1 means that the *i*th feature is selected into the subset *U*, and *f_i_* = 0 means the *i*th feature is not selected. The objective function of the proposed feature selection algorithm can be defined as:

(3) $$\begin{array}{l} \underset{f}{\text{max}}\left(\frac{f^{T}S}{k}-\frac{f^{T}Cf}{k(k-1)}\right)\\ s.t.\sum_{i}f_{i}=k,f_{i}\in \{0,1\} \end{array}$$

In Equation (3), $\frac{f^{T}S}{k}$ stands for the average weight of the selected features, $\frac{f^{T}Cf}{k(k-1)}$ stands for the average correlation among the selected features, and the constraints are used for restricting the number of selected features in the *U* to be *k*. Thus, maximizing Equation (3) can ensure that the selected features in *U* are most important and least redundant. However, Equation (3) is a quadratic integral programming problem and it is hard to be solved [32]. Therefore, in our study, we relax the constraint of *f_i_* ε{0,1} *f_i_* to ε[0,1], and convert the objective function in Equation (3) to:

(4) $$\begin{array}{l} \underset{f}{\text{max}}\left(\frac{f^{T}S}{k}-\frac{f^{T}Cf}{k(k-1)}\right)\\ s.t.\sum_{i}f_{i}=k,f_{i}\in [0,1] \end{array}$$

### 3.3. Solution

In this section, a pair-wise updating algorithm similar to that found in [32] is introduced to solve the maximization problem in Equation (4).

The Lagrangian function of Equation (4) can be derived as:

(5) $$L(f,\lambda ,\mu ,\beta )=\left(\frac{f^{T}S}{k}-\frac{f^{T}Cf}{k(k-1)}\right)-\lambda \left(\sum_{i}f_{i}-k\right)+\sum_{i}\mu_{i}f_{i}+\sum_{i}\beta_{i}(1-f_{i})$$

Where λ, μ*_i_* and β*_i_* are Lagrangian multipliers. Based on the Karush-Kuhn-Tucker (KKT) conditions [33], the solution that maximizes the Equation (4) must satisfy the first-order necessary conditions as:

(6) $$\{\begin{array}{l} {\left(\frac{S}{k}-\frac{2Cf}{k(k-1)}\right)}_{i}-\lambda +\mu_{i}-\beta_{i}=0\\ \sum_{i}\mu_{i}f_{i}=0\\ \sum_{i}\beta_{i}(1-f_{i})=0 \end{array}$$

where ${\left(\frac{S}{k}-\frac{2Cf}{k(k-1)}\right)}_{i}$ is the *i*th element of vector $\frac{S}{k}-\frac{2Cf}{k(k-1)}$. Because *f_i_*, μ*_i_* and β*_i_* are all non-negative, $\sum_{i}\mu_{i}f_{i}=0$ means that if *f_i_* > 0, then μ*_i_=* 0. Similarly, $\sum_{i}\beta_{i}(1-f_{i})=0$ means that if *f_i_* < 1, then β = 0. Thus, according to the relationship between ${\left(\frac{S}{k}-\frac{2Cf}{k(k-1)}\right)}_{i}$ and λ, the KKT conditions can be rewritten as:

(7) $${\left(\frac{S}{k}-\frac{2Cf}{k(k-1)}\right)}_{i}\{\begin{array}{ll} \leq \lambda & f_{i}=0\\ =\lambda & f_{i}\in (0,1)\\ \geq \lambda & f_{i}=1 \end{array}$$

Here, since ${\left(\frac{S}{k}-\frac{2Cf}{k(k-1)}\right)}_{i}$ could reflect the relationship between the feature’s weight and its average correlation with other features in *U*, we call it the reward of *i*th feature, and denote it by *r_i_*(*f*). According to the value of ${\left(\frac{S}{k}-\frac{2Cf}{k(k-1)}\right)}_{i}$, we can partition the feature set into three subsets, *U_1_*= {*P_i_* | *f_i_*=0}, *U_2_*= {*P_i_* | *f_i_*ε(0,1)} and *U_3_*= {*P_i_* | *f_i_*=1}. From the constraints of *f* in Equation (4), it can be found that if a feature is in subset *U_1_* or *U_2_*, the value of its corresponding element in *f* can be increased. On the contrary, if a feature is in subset *U_2_* or *U_3_*, the value of its corresponding element in *f* can be decreased.

The pair-wise updating strategy to solve Equation (4) is defined as:

(8) $$f_{l}^{new}=\{\begin{array}{ll} f_{l} & l\neq i,l\neq j;\\ f_{l}+\alpha & l=i;\\ f_{l}-\alpha & l=j; \end{array}$$

That is, only the values of two elements in *f* (*f_i_* and *f_j_*, *i* ≠ *j* ) are updated in each iteration of our algorithm. After updating *f_i_* and *f_j_*, the change of Equation (4) is:

(9) $$\begin{array}{l} \Delta =\left(\frac{f^{newT}S}{k}-\frac{f^{newT}{Cf}^{new}}{k(k-1)}\right)-\left(\frac{f^{T}S}{k}-\frac{f^{T}Cf}{k(k-1)}\right)\\ =\frac{f^{newT}S-f^{T}S}{k}+\frac{f^{T}Cf-f^{newT}{Cf}^{new}}{k(k-1)}\\ =\frac{(s_{i}-s_{j})\alpha }{k}+\frac{(2C_{ij}-C_{ii}-C_{jj})\alpha^{2}+2(e_{j}Cf-e_{i}Cf)\alpha }{k(k-1)}\\ =\frac{(2C_{ij}-C_{ii}-C_{jj})\alpha^{2}+(k-1)(s_{i}-s_{j})\alpha +2(e_{j}Cf-e_{i}Cf)\alpha }{k(k-1)} \end{array}$$

where *e_i_* is a row vector with only the *i*th element equal to 1, and 0 otherwise. So, Equation (9) can be further converted as:

(10) $$\begin{array}{l} \Delta =\frac{(2C_{ij}-C_{ii}-C_{jj})\alpha^{2}}{k(k-1)}+\frac{(k-1)s_{i}\alpha -2e_{i}Cf\alpha }{k(k-1)}-\frac{(k-1)s_{j}\alpha -2e_{j}Cf\alpha }{k(k-1)}\\ =\frac{(2C_{ij}-C_{ii}-C_{jj})\alpha^{2}}{k(k-1)}+\left(\frac{s_{i}}{k}-\frac{2e_{i}Cf}{k(k-1)}\right)\alpha -\left(\frac{s_{j}}{k}-\frac{2e_{j}Cf}{k(k-1)}\right)\alpha \\ =\frac{(2C_{ij}-C_{ii}-C_{jj})\alpha^{2}}{k(k-1)}+{\left(\frac{S}{k}-\frac{2Cf}{k(k-1)}\right)}_{i}\alpha -{\left(\frac{S}{k}-\frac{2Cf}{k(k-1)}\right)}_{j}\alpha \\ =\frac{(2C_{ij}-C_{ii}-C_{jj})\alpha^{2}}{k(k-1)}+\left(r_{i}(f)-r_{j}(f)\right)\alpha \end{array}$$

With the aim of maximizing Δ, according to Equation (10) and the constraints of *f*, α can be computed as:

(11) $$\alpha =\{\begin{array}{ll} \text{min}(f_{j},1-f_{i}) & \text{if }2C_{ij}-C_{ii}-C_{jj}\geq 0\;\text{and }r_{i}(f)>r_{j}(f)\\ \text{min}(f_{j},1-f_{i},\frac{k(k-1)\left(r_{j}(f)-r_{i}(f)\right)}{2C_{ij}-C_{ii}-C_{jj}}) & \text{if }2C_{ij}-C_{ii}-C_{jj}<0\;\text{and }r_{i}(f)>r_{j}(f)\\ \text{min}(f_{j},1-f_{i}) & \text{if }2C_{ij}-C_{ii}-C_{jj}>0\;\text{and }r_{i}(f)=r_{j}(f) \end{array}$$

Note that in the updating algorithm above, only the situation that *r_i_*(*f*) ≥ *r_j_*(*f*) is considered. If *r_i_*(f) < *r_j_*(*f*), exchange *i* and *j* to implement the algorithm.

By iteratively updating the values of pair-wise elements in *f* and computing α using Equations (8) and (11), the objective function in Equation (4) can be increased and reach its maximum [32]. The implementation details of the proposed feature selection method are summarized in Algorithm 1.

As can be seen in Algorithm 1, a heuristic strategy is adopted in each iteration of the pair-wise updating algorithm to increase the objective function maximally. In this strategy, a pair of elements in *f* whose values should be updated is selected according to the rewards of their corresponding features. In other words, the element whose value should be increased in each iteration is selected as the one whose corresponding feature has the largest reword in subset *U_1_* or *U_2_*, and the element whose value should be decreased in each iteration is selected as the one whose corresponding feature has the smallest reword in subset *U_2_* or *U_3_*. From Equation (10), we can find that the increase of the objective function in Equation (4) can be maximized by this method. The solution of proposed algorithm is obtained when the value of Equation (4) cannot be further increased.

## 4. Conclusions

In this study, a novel feature selection method based on maximum weight and minimum redundancy criterion is proposed. Comprehensive experiments and deep analysis based on the microporous aluminophosphates (AlPOs) database demonstrate the effectiveness of the proposed algorithm. This work also demonstrates the feasibility of feature selection techniques in chemical data analysis. By taking advantage of the proposed algorithm, we investigate the relationship between synthetic factors and rational synthesis of microporus materials. The classification result with a classification accuracy rate of 91.12% shows that a number of synthetic factors including the molar amount of Al_2_O_3_, the molar amount of solvent, the molar amount of template in the gel composition, the melting point, the dipole moment, the second longest distance of organic template, the dipole moment, the ratio of *C/N*, the ratio of **N***/*(*C* + *N*), the ratio of *N*/Van der Waals volume and the maximal number of protonated H atoms play vital roles for rational synthesis of (6,12)-ring-containing AlPOs. Among these optimal synthetic factors, the second longest distance of organic template, which is the geometric size of the organic template, plays the most important role in the prediction. This work provides *a priori* knowledge and a useful guidance for rational synthesis experiments of such materials.

In future studies, we will gradually add more synthetic features (or factors) into the database to investigate their influences for the synthesis of AlPOs.

## Figures and Tables

**Table 1:** Confusion matrix.

Hypothesis	Actual positive	Actual negative
Hypothesise positive	True positive (*TP*)	False positive (*FP*)
Hypothesise negative	False negative (*FN*)	True negative (*TN*)

**Table 2:** Highest classification accuracy rates reached by the original and the proposed feature selection methods.

	Nearest Neighbor		Naive Bayes	
		
Method	Highest Acc_Rate	Dimension	Highest Acc_Rate	Dimension
F	0.9080	20	0.8736	3
FI	0.9112	12	0.8767	5
R	0.9088	17	0.8608	21
RI	0.9096	17	0.8608	3
G	0.9080	21	0.8624	19
GI	0.9096	13	0.8648	13

**Table 3:** Highest *F*-measure reached by the original and the improved feature selection methods.

Method	Highest *F*-measure (Nearest Neighbor)	Highest *F*-measure (Naive Bayes)
F	0.8144	0.7817
FI	0.8586	0.8071
R	0.8585	0.7851
RI	0.8599	0.7851
G	0.8518	0.7640
GI	0.8579	0.8003

**Table 4:** Highest classification accuracy rates reached by different feature selection methods.

	Nearest Neighbor		Naive Bayes	
		
Method	Highest *Acc_Rate*	Dimension	Highest *Acc_Rate*	Dimension
Constraint Score	0.908	21	0.8639	19
Ttest	0.9096	19	0.8728	2
FCBF	0.8072	/	0.8584	/
MRMR	0.908	21	0.868	1
Our algorithm (FI)	0.9112	12	0.8767	5

**Table 5:** Optimal *F*-measure values reached by different feature selection methods.

Method	*F*-measure (Nearest Neighbor)	*F*-measure (Naive Bayes)
Constraint Score	0.8388	0.7588
Ttest	0.8046	0.7825
FCBF	0.5416	0.7730
MRMR	0.7723	0.7721
Our algorithm (FI)	0.8586	0.8071

**Table 6:** Description of the input synthetic factors.

Category	ID	Description
Gel composition	F1	The molar amount of Al_2_O_3_ in the gel composition
	F2	The molar amount of P_2_O_5_ in the gel composition
	F3	The molar amount of solvent in the gel composition
	F4	The molar amount of template in the gel composition

Solvent	F5	The density
	F6	The melting point
	F7	The boiling point
	F8	The dielectric constant
	F9	The dipole moment
	F10	The polarity

Organic template	F11	The longest distance of organic template
	F12	The second longest distance of organic template
	F13	The shortest distance of organic template
	F14	The Van der Waals volume
	F15	The dipole moment
	F16	The ratio of *C/N*
	F17	The ratio of *N/*(*C* + *N*)
	F18	The ratio of *N*/Van der Waals volume
	F19	The Sanderson electronegativity
	F20	The number of free rotated single bond
	F21	The maximal number of protonated H atoms

**Algorithm 1:** The feature selection process of the proposed method.

Input: The original data sample *D*. Output: The indicator vector *f*. 1. Compute scores of features *S* and correlation matrix *C*. 2. Initialize *f*; 3. Do 4. Select *P_i_* ε *U*_1_ ∪ *U*_2_ which has the largest reward *r_i_*(*f*); 5. Select *P_j_* ε *U*_2_ ∪*U*_3_ which has the smallest reward *r_j_*(*f*); 6. if *r_i_*(*f*) > *r_j_*(*f*) Compute α using Equation (11), and then update *f_i_* and *f_j_* according to Equation (8); 7. else if *r_i_*(*f*) = *r_j_*(*f*) 8. if 2*C_ij_* − *C_ii_* − *C_jj_* > 0 Compute α using Equation (11), and then update *f_i_* and *f_j_* according to Equation (8); 9. else if 2*C_ij_* − *C_ii_* − *C_jj_* = 0 Check whether there exist a *P*_0_ ε *U*_1_ ∪ *U*_2_ and a *P_x_* ε *U*_2_ ∪ *U*_3_ such that 2*C_ox_* − *C_oo_* − *C_xx_* > 0 and *r_o_*(*f*) = *r_x_*(*f*). If the pair (*P_o_*, *P_x_*) can be found, Compute α using Equation (11), and then update *f_o_* and *f_x_* according to Equation (8); Otherwise, *f* is a solution of Equation (4); 10. end if 11. end if 12. until *f* is a solution of Equation (4).
